# Parallel perspectives for building sustainable safety initiatives

**DOI:** 10.1002/acm2.12690

**Published:** 2019-07-31

**Authors:** Eric C. Ford, Jean M. Moran, Gwe‐Ya Kim, Leah Schubert, Yi Rong

**Affiliations:** ^1^ Department of Radiation Oncology University of Washington Seattle Washington; ^2^ Radiation Oncology University of Michigan Ann Arbor Michigan; ^3^ Radiation Medicine and Applied Sciences University of California, San Diego San Diego California; ^4^ Radiation Oncology University of Colorado School of Medicine Aurora Colorado; ^5^ Radiation Oncology University of California Davis Sacramento California

## 1 INTRODUCTION

Safety improvement is just that, a continuous improvement toward safer and higher quality operations. As such, it is not a destination that one will reach, after which no further effort is needed. Rather it is a continuous process that must go on as long as patients are to be treated. Viewed this way, the most important aspect of a safety and quality program is the long‐term investments toward sustainability. What are the programmatic initiatives and actions that can be taken now to foster a successful program for years to come? The role of medical physicist in quality and safety for clinical environment and its impact on the medical physics profession has been fiercely discussed in the previous editorials.^1^, ^2^ This article includes perspectives from four thought‐leaders on achieving sustainable programs in safety and quality considering resources, leadership, sharing ownership and participating in quality improvement (QI) efforts, integrating with daily work, selecting a focus for projects, and celebrating wins as a team.

Eric Ford, PhD, FAAPM is Professor and Interim Director of Medical Physics at University of Washington (UW), Seattle. Dr. Ford was trained at Memorial Sloan‐Kettering and was on the faculty of Johns Hopkins before moving to UW. His research on safety and quality has helped inform the understanding of risk analysis and safety in radiation oncology and he has been instrumental in the development of the national system RO‐ILS: Radiation Oncology Incident Learning System sponsored by American Association of Physicists in Medicine (AAPM) and American Society for Radiation Oncology (ASTRO). Dr. Ford serves on the AAPM Board of Directors. He will be co‐director of the 2020 AAPM Summer School “Advances in Quality Assurance.

Dr. Jean Moran is Professor and Co‐Director of Physics at the University of Michigan and Associate Director of the Michigan Radiation Oncology Quality Consortium. She serves as Chair of the AAPM's Therapy Physics Committee and the Work Group on Radiation Oncology — Incident Learning Systems. She has been involved in numerous safety and quality‐related activities in AAPM and ASTRO. She led the creation and implementation of the Safety Sunday on the Therapy Track of the AAPM Spring Clinical Meeting from 2014 to 2017. She is passionate about integrating patient safety in daily work, education, and research activities to benefit our patients.

Dr. Grace Gwe‐Ya Kim is an Associate Professor and Associate Division Director of Quality and Safety in the Department of Radiation Medicine and Applied Sciences at the University of California, San Diego. She is an active AAPM member and is currently serving on the Working Group (WG) on Prevention of Errors in Radiation Oncology as Chair, and is a member of WG Radiation Oncology Incident Learning System (ROILS), TG262, TG275, and TG327 etc. Her research focuses on implementing novel treatment technique, patient safety, and improvements in intracranial radiosurgery procedures.

Leah Schubert, Ph.D. DABR is the Associate Professor in the Department of Radiation Oncology and Program Director of the Medical Physics Residency Program at the University of Colorado School of Medicine. She has co‐chaired her department’s Quality and Safety Committee since its inception in 2012 and has been involved in numerous quality improvement projects. Dr. Schubert co‐led the development of incident learning across the radiation oncology departments in the University of Colorado Health System. She is a member of the AAPM Workgroup on Prevention of Errors in Radiation Oncology.

## 2 ERIC FORD, PH.D. — LEADERS’ ROLES IN SAFETY IMPROVEMENT

In my view, safety and quality improvement are not an optional “extra” added on by the physicist but rather a key core feature of operations in any medical clinic. Few would argue with this, and in fact in radiation oncology, this is baked into the practice accreditation standards. However, in my experience, there is a wide variety in the rigor, quality, and structure of such programs. Why? The answer, that I have come around to, is that much of this is driven by leadership. Even a few strong negative words from someone in a leadership can have a lasting chilling effect, while the opposite is also true.

In considering quality and safety, several questions arise in particular around the role of leaders. What are the responsibilities of a leader? How can leaders promote high‐quality safety and quality improvement programs? What are the key features of a high‐quality program that leaders can (should) promote?

### 2.1 Leaders: Who are they? Do they “get it”?

Leadership is exerted on many levels. Most centers have formalized leadership roles (department chair, clinical directors, educational director, etc.) and these leaders have the potential to allocate resources, organize effort, call meetings, and the like. However, leadership is exerted in many ways and medical physicists are seen as leaders in the clinic whether they wear such a title or not.

Some of these leaders (whoever they are) naturally appreciate the importance of rigorous safety and quality improvement programs. Others less so. However, a passive stance with respect to safety and quality programs is becoming increasingly untenable. Published guidelines now make pointed recommendations for a formalized program. This includes a series of white papers from ASTRO and the “Safety is No Accident” report (recently updated and republished this year),^3^ practice standards from American College of Radiology (ACR), ASTRO and American Society of Radiologic Technologists (ASRT),^4^, ^5^ and requirements for practice accreditation by ACR, American College of Radiation Oncology (ACRO) and ASTRO^6^ (note that approximately half of practices in the US are accredited and some states require it). In addition, there are similar recommendations from other national and international bodies such as International Atomic Energy Agency (IAEA) and World Health Organization (WHO). Most importantly, leaders are answerable to patients who expect and deserve high‐quality care.

### 2.2 Resources and structure

One common struggle in safety and quality improvement programs is the effort required to implement and maintain such a program. This is a common theme that I often hear at meetings and in discussions with medical physicists. This is an area where leadership can potentially act directly through allocation of resources and staff effort. Though the importance of resource allocation should not be underestimated, it should be recognized that even a highly motivated leader may experience limits to what can be done directly. In a clinic with two medical physicists, for example, it may be unlikely that a third can be hired in order to implement and manage a safety and quality improvement program.

There are, however, many dimensions of an organization that leadership can influence beyond resource allocation. Perhaps most importantly the leader can establish and maintain the structure of a program that intelligently employs effort. Consider the operations of an incident learning system. Such a system can require substantial effort.^7^ However, the bulk of this effort need not be shouldered by the medical physicist nor, arguably, should it be. The effort should be distributed amongst many professional staff and trainees. In this way the effort of any one person can be kept manageable. Leadership plays a key role in making this happen. An active and engaged leader can implement a structure to the program, set expectations around it, and ensure its continued operation.

### 2.3 Sustainability and engagement

Directly related to this is the sustainability of a safety and quality improvement program. To have the maximum impact on quality of care for patients, the program must be viewed as more than “one‐off” projects designed to address a specific issue. It must be sustainable for many years. There are some key aspects of this which can be promoted by leaders.

Engagement of all staff is a key requirement. Feedback is a crucial driver of this. If people see issues being addressed in a positive way and observe that processes and culture are changing for the better then engagement grows. At least one study has shown this quantitatively, that is, when staff get more feedback engagement grows.^8^ Other studies in our literature have measured culture changes over time^9^, ^10^, ^11^ and shown that they can be sustainable.^12^

One key to building engagement is to make safety and quality improvement a daily activity or near daily activity. For medical physicists, this is natural since many of the job tasks are directly related to quality and safety. It is, however, more difficult to engage on programmatic‐level safety and quality initiatives with other staff, at least not on a regular or very regular basis. This is one of the disadvantages of risk analysis projects relying on failure modes and effects analysis (FMEA) and fault tree analysis (FTA) *a la* AAPM Task Group 100 and the like.^13^ While it may be possible to perform these regularly (our clinic for example does these twice per year), these exercises are typically not done very frequently, certainly not daily.

### 2.4 Summary

Effective quality and safety programs engage all staff and can be maintained over years. The role of leadership is key in making this happen and all medical physicists are leaders. Through such structured programs we can ensure safe and high‐quality care for our patients.

## 3 JEAN M. MORAN, PH.D. — INTEGRATING QUALITY AND SAFETY INTO DAILY WORK

I have been fortunate to work with leaders, mentors, and colleagues who unabashedly support quality and safety initiatives as a part of our daily care for patients. After almost 30 years in different roles in an academic radiation oncology department, I have witnessed the value of teamwork for improving quality and safety. We use formal incident reporting and learning to energize and guide our efforts. My colleagues and I are fully committed to our patients regardless of whether we are providing direct service to patients or supporting our mission via scheduling patients, providing administrative support, or engineering better software tools.

### 3.1 Department and institutional climate

Our department and hospital missions enthusiastically value safety and quality for patient care. We regularly use different tools in our shared toolbox to ensure the longevity of QI in our daily clinical lives. Many of our initiatives focus on ensuring the intended outcome for our patients while reducing waste in processes. We have successfully applied lean practices to different projects, beginning with a project to get patients with bone and brain metastases safely and promptly under treatment.^14^ Our 2018 annual department (main campus and community practice clinics) retreat focused on retraining and reinvigorating our lean efforts.

Keeping an eye on our department resources is important. After monitoring our responses to incidents and ability to follow‐up, our leadership team took the innovative step of hiring a dedicated Quality and Safety Officer. We designed the position to be a department and community practice resource. Katie Woch Naheedy, our Quality and Safety Officer, is a member of our department Operations Team so that feedback can be provided on both ongoing operations and new initiatives. She regularly presents to different groups in our department and at multidisciplinary meetings about incidents in our clinic. She also leads our reporting in the ASTRO AAPM RO‐ILS^15^ and facilitates open discussions based on RO‐ILS aggregate reports to assess our risk of similar events.

### 3.2 The value of continuous learning

A learning mindset is crucial when it comes to improving patient safety and there are many opportunities for training. Further education is invaluable, such as institutional training in lean techniques for health care, AAPM training on using the tools of Task Group 100,^13^ or the application of formal root cause analysis. We leverage medical school resources by having individuals attend team thinking training and through participation in the University of Michigan Quality Leadership Scholars program (PASQUAL).^16^

When someone participates in training, we encourage application of any new knowledge in the clinic to benefit our patients and the learner. For example, after learning about the formal root cause analysis (RCA) methods used by the VA National Center for Patient Safety (NCPS), we have adopted RCAs into our department process following the VA NCPS guide.^17^ We include our trainees as team members so that using formal QI tools becomes a second nature to our next generation.

### 3.3 Integrating quality and safety into daily work

In our clinic, supporting patient safety is a team sport where we all play, participate, and strive toward improvement. Supporting safety means focusing on processes not people, and ensuring a just culture.^18^ This is woven into the fabric of our day. To make efforts sustainable, members must respect each other, be fully committed to understanding where the data direct us, and be consistent in the implementation for workflow changes. We sometimes couple clinical care with research interests to solve challenging and intriguing problems. Many of us in medical physics were drawn to the opportunity to boldly face challenges in support of patients and their loved ones. Two example safety improvements are described below.

#### 3.3.1 Example challenge

Our peer review process for stereotactic body radiation therapy patients took place after plans were already created. If a change was needed, there was significant rework by the attending physician, dosimetrist, and physicists.

#### 3.3.2 Solution

Move peer review to the beginning of the process, measure the effectiveness of the intervention, and then share the results for open discussion with the department.^19^

#### 3.3.3 Example challenge

Despite using automation to support our physics plan checks,^20^ errors generated during treatment planning were still reaching the treatment unit.

#### 3.3.4 Solution

Work as a team to analyze the types and frequency of errors reaching the treatment unit, design an intervention (therapist prestart treatment plan QA), measure the effectiveness, and share the results for open discussion with the department.^21^

### 3.4 Summary

We have a track record of applying and quantifying the effectiveness of formal QI tools in our clinic. These efforts are well served by employees who are persistent, grounded in a commitment to patients, and flexible when different approaches are needed. It has made a positive impact that these are department‐wide initiatives where everyone has a place at the table.

## 4 GRACE GWE‐YA KIM, PH.D. — ESTABLISHING AN OVERSEEING SAFETY COMMITTEE

A departmental incident learning program in Radiation Medicine & Applied Sciences, UC San Diego, was established and has been active in promoting safety and accident prevention since 2010. As the chair of the safety committee, I am honored to participate in the departmental safety initiatives where I facilitate and moderate the investigation and discussion of possible solutions for safety problems reported in our incident learning system. We have experienced many years of great growth and development through multiple safety and quality improvement initiatives. Some of the experiences at our center were published in the AAPM Newsletter through an interview of our Director of Physics, including tips and tricks of leading quality and safety initiatives in the clinic as a perspective of leadership.^22^ Here are the lessons I have learned from my perspective in the field, as well as a couple of points that I want to emphasize to help others create sustainable safety systems.

One important measure is the creation of a safety committee with clear responsibilities. This committee oversees compliance with standards and regulations to support the efficiency of the rest of the clinical team. To regulate safety successfully, the team has to be committed to the welfare of the department and work collectively to ensure the safety and performance of the system. A sense of trust between the safety committee members and all employees is required to enable confidence in the work that is being done. For a high functioning safety team, it is beneficial for each member to invest time and effort to analyzing incidents and sharing their perspectives on the expected effectiveness of the recommended precautions in response to an event. These precautions recommended by the committee must be clear to all members of the department. Members of the safety committee may provide additional oversight in the safety and regulation of the system’s performance and supporting necessary tasks to achieve that goal. These tasks may include integrating different safety measures and researching various approaches for improving patient care. The safety committee could also be responsible for managing formal incident reporting to maximize the safety and health of the patients. The committee can support safety using incident reporting by holding discussions and notifying the entire department of the incidents that have occurred. These meetings would alert the team members about the risks and encourage broader engagement when brainstorming solutions. This is an efficient way of keeping everyone up to date and improving communication within the department to maximize safety.

### 4.1 The structure of the safety committee

By creating a clear structure with defined goals and responsibilities, the safety committee will be better positioned to address even large quantities of incident reports. Frequent reports of the same type of incident or near miss reports represent a good safety culture of reporting any and all incidents. However, reporting safety concerns alone is not enough. It is beneficial for the safety committee, using a well‐defined strategy, to produce in‐depth analyses and when appropriate strong interventions to reduce risks to patient safety and lead to meaningful change in the clinical environment.

### 4.2 Necessary funding and support

Managing an incident learning program should be considered an investment. Leaders and managers need to provide adequate tools and resources for sustainable ongoing improvement projects. Systems will be less effective if they lack financial support, have a shortage of dedicated effort, expertise, and time, or inadequate training to deal with the high volume of reports. The leadership’s commitment is essential for a robust incident learning program. The safety committee members should be empowered to actively engage with staff and reallocate resources to enable intervention‐based causal analysis to ensure safety. Providing feedback after the submission of the incident report helps team members see the value of reporting and can lead to even more reporting in the future.

### 4.3 Broad participation

All members in the department should be involved, and it must be impressed upon them exactly what is needed to maximize each patient’s safety. However, the safety committee may often face challenges to finding ways to involve all staff. Under‐reporting makes incident reporting less useful because the reports that are submitted provide a biased snapshot of issues. Multiple publications or reports demonstrate an imbalance in reporting where certain professional group submits a relatively high volume of the adverse report compared to other groups. For example, physicians tend not to report events which limit learning opportunities for the department. Mitchell et al.^23^ reported doctors mistrust patient safety reporting because they are afraid of how the reports will be used, uncertainty about what to report, do not receive feedback, and see action resulting from reporting. We must continue to work to encourage all employees to participate in incident reporting and supporting patient safety.

### 4.4 Summary

The incident learning program is an important system to prevent adverse events, provide an opportunity to improve workflow and promote safety culture in the department. The integration of a well‐defined strategy of the safety committee, adequate resource allocation, and the broad participation of all members in the department are essential components in building a sustainable safety program.

## 5 LEAH K. SCHUBERT, PH.D. — MAINTAINING PRODUCTIVITY AND ENGAGING STAFF FOR SUSTAINABLE QUALITY IMPROVEMENT

Small steps can help you travel long distances. The underlying goal of safety and quality programs is to motivate change that will result in improvement of patient care, but these changes can be difficult to achieve in daily practice. However, if changes are not ultimately made, staff can lose motivation and a culture of apathy can set in. Thus making even small, incremental changes can help engage staff and is necessary for building a program that is ready to address new risks that may arise. Our department has found techniques over the years to facilitate productivity and motivate staff participation, which are discussed below.

### 5.1 Support from leadership and staff

The primary factor for sustaining our program has been the dedication of leadership and staff. Our program has been supported by, not only the Department Chair and Chief Physicist, but also by leaders of all staffing groups in our department. Recognizing the important role of each staff member and allowing them to voice concerns has helped build a culture of trust and transparency. Staff members also support safety efforts through the use of our incident learning system, which is the driving system of our safety and quality program. Following up with staff on reports and communicating the changes made in response to their original concerns is important for sustaining staff engagement. “It takes a village” to improve safety.

### 5.2 Leveraging existing resources

This saying not only applies to our department, but to other medical specialties and organizations such as the AAPM. These days most hospitals have existing quality and safety resources available for use. Many of our faculty, staff, and residents have received excellent training through our hospital’s Institute of Healthcare Quality Safety and Efficiency. The more members of the team who are knowledgeable in the principles of quality improvement and the rationale behind initiatives, the more likely it is to have staff agree and comply with initiatives. On a national level, the AAPM virtual library provides access to resources from specialty meeting and numerous conference education sessions. Simply talking to AAPM members, asking questions and getting their insights, can be tremendously influential. These experts have generously shared their experiences with their own programs in order to help us build ours.

### 5.3 Productive committee with motivated staff

In order to ensure progress toward safety improvement, we created a quality and safety committee within our department. A physician chair and physics chair share responsibility for the committee’s productivity and encourage involvement from department faculty. The members of the committee actively combine their efforts and are dedicated to making improvements. The committee is also open to all staff, including residents and other trainees, in an effort to engage staff and create transparency of processes.

Holding productive meetings is a key component for progress toward safety improvement. The multidisciplinary and continuous nature of safety improvement involves asking many busy people to spend time in frequent meetings. Everyone’s time is valuable, so these meetings need to be productive. Our meetings are goal‐based and fast‐paced. Committee members volunteer and take ownership of a particular improvement, which helps to distribute the workload. We also form smaller subcommittees to focus on more complex problems, who then report back to the full committee. This optimizes time spent at the full committee meetings.

### 5.4 Being selective in our efforts

Frequent meetings are needed to keep pace with incident reports and continuous suggestions for improvement. While there is the constant drive to try to fix everything, being realistic and selectively choosing what improvements to implement is crucial. Focusing on small wins and “chunking” larger projects can allow for successful larger system process changes. When sustainability is the end goal, it is important to take into account the following considerations: (a) weighing the need for a particular improvement, (b) determining whether the improvement will be effective, (c) balancing against the effort required for implementation and likelihood of staff compliance, and (d) understanding the staff and department bandwidth for change.

### 5.5 Focusing on the positive and celebrating wins

Safety improvement often starts with a problem, which can easily set a negative tone. Rather than dwelling on the negatives, focusing on the positives has been instrumental. This takes conscious effort from committee leaders. A nonpunitive approach in which the focus is on improvements helps with staff engagement. Establishing a positive, energized atmosphere in meetings also cultivates brainstorming. Recognizing staff for the good catches that they make is always well received and constructively broadens knowledge of near misses. Additionally, our department hosts a Safety Month every year. While it started out as a means to conduct required emergency training, it has evolved into a broad celebration of safety efforts that provides a vehicle to communicate appreciation of staff members’ continuous efforts toward safety improvement.

### 5.6 Summary

Maintaining productivity and engaging staff are key ingredients in achieving sustainable safety and quality programs. A quality and safety committee focused on thoughtfully making improvements, no matter how small and incremental, can distribute the workload, and motivate staff participation. Celebrating team wins can broadly communicate the continuous efforts that all staff members make to improve safety.

## CONFLICTS OF INTEREST

The authors declare no conflicts of interest.
